# Immune Checkpoint Inhibitor‐Induced Pancreatitis

**DOI:** 10.1002/ueg2.70285

**Published:** 2026-09-07

**Authors:** Carlos Fernandez Moro, Atsuko Kasajima, Suzane Ribeiro, Linnéa Molander, Celina Limbecker, Miroslav Vujasinovic, Günter Klöppel, Anne Hoorens, Caroline S. Verbeke, J.‐Matthias Löhr

**Affiliations:** ^1^ Department of Laboratory Medicine Division of Pathology Karolinska Institutet Stockholm Sweden; ^2^ Department of Clinical Pathology and Cancer Diagnostics Karolinska University Hospital Stockholm Sweden; ^3^ Department of Pathology TUM School of Medicine and Health Technical University Munich Munich Germany; ^4^ Department of Gastroenterology Ghent University Hospital Ghent Belgium; ^5^ Department of Clinical Science Intervention and Technology Karolinska Institutet Stockholm Sweden; ^6^ Department of Medicine Huddinge Karolinska Institutet Stockholm Sweden; ^7^ Department of Upper Digestive Diseases Karolinska University Hospital Stockholm Sweden; ^8^ Department of Pathology Ghent University Hospital Ghent Belgium; ^9^ Department of Pathology Oslo University Hospital Oslo Norway; ^10^ Institute of Clinical Medicine University of Oslo Oslo Norway

**Keywords:** autoimmune pancreatitis, immune checkpoint inhibitor, immune‐related adverse events

## Abstract

The increasing use of immune checkpoint inhibitors (ICI) has led to recognition of a broad spectrum of treatment‐associated inflammatory adverse events, including pancreatic injury. Histological overlap between ICI associated pancreatic injury (ICIPI) and so‐called autoimmune pancreatitis (AIP) has been suggested in isolated reports, but the extent and detailed histopathological features of this overlap remain poorly characterized. The aim of this study was to describe the clinicopathological and histological features of rare ICIPI cases with available tissues and to compare these findings with established histological patterns of AIP. Data were available for five cases. All patients except one were female. ICIPI occurred between 182–580 days after initiation of ICI therapy. All patients had received a combination ICI treatment (e.g., Ipilimumab + Nivolumab). Peak serum lipase levels ranged from 4 to 41 (µkat/L). Computed tomography demonstrated radiologic features consistent with autoimmune pancreatitis (AIP) in four cases, whereas one case raised suspicion of a metastatic lesion. Three patients were treated with steroids, following which pancreatitis (lipase and imaging) resolved. Two patients developed pancreatic atrophy or exocrine insufficiency. Two patients underwent surgical resection because of suspected cancer metastasis. Fine needle biopsy showed only nonspecific atrophy and mild chronic inflammation. In contrast, both surgical specimens demonstrated marked histological overlap with type 2 AIP, including duct‐centric inflammatory changes and granulocytic epithelial lesions, whereas increased IgG4‐positive plasma cells or other features characteristic of type 1 AIP were not identified. Our results, together with the limited published literature, suggest that ICIPI may encompass a heterogeneous spectrum of histological changes, with substantial overlap with type 2 AIP in a subset of cases. Recognition of these overlapping features is important in the histopathological diagnosis of pancreatic inflammatory lesions in patients receiving ICI therapy. Treatment of ICIPI should follow NCCN recommendations. The risk of developing a pancreatic exocrine insufficiency needs to be observed.

## Key Summary

- Summarize the established knowledge on this subject.
  ◦ The immune checkpoint inhibitor associated pancreatic injury (ICIPI) is a rare but typical side effect of ICI therapy
  ◦ ICIPI is considered a form of autoimmune pancreatitis
  ◦ Most cases of ICIPI are mild and are treated according to NCCN guidelines
- What are the significant and/or new findings of this study?
  ◦ The histology of ICIPI is not homogenous but includes features of both type 1 AIP and even more so type 2 AIP
  ◦ A thorough histopathological typing can only be established from surgical pathology but not be deduced from FNB
  ◦ Histopathology does not allow to call ICIPI a distinct third type of autoimmune pancreatitis
  ◦ As with other forms of autoimmune pancreatitis, there is a high likelihood of pancreatic atrophy and subsequent pancreatic exocrine insufficiency.

## Graphical abstract

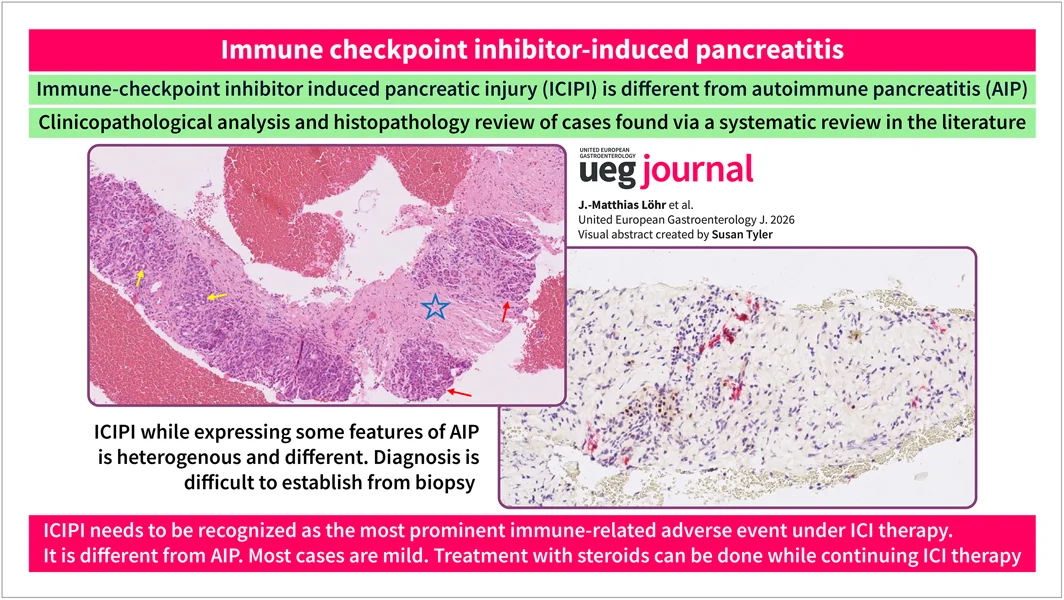

## 1 Introduction

Immune checkpoint inhibitors (ICI) work by blocking CTLA‐4 or PD‐1/PD‐L1, consequently preventing the “off” signal and allowing T‐cells to remain active and kill tumor cells. Given the success in treating malignancies, ICIs are currently part of treatment for many solid cancers, leading to the term “PDLomas” [1]. However, a broad spectrum of immune‐related adverse events associated with immune checkpoint blockade (ICI‐irAE) [2] has been reported, which are likely the result of increased activity of the host immune system causing inflammatory side effects [3], in part by reactivating autoimmune disease known to be associated with PD‐1/CTLA‐4 polymorphisms [1] or to unleash pre‐existing autoantibodies [4, 5]. While any organ can be affected, irAEs most commonly involve the gastrointestinal tract, including the pancreas [2, 3]. The incidence of ICI associated pancreatic injury (ICIPI) is reported to be between 1% and 5%, depending on whether a single ICI is used or a combination thereof [6]. Cases of severe acute pancreatitis are rare [6].

Because some clinical and radiological features of ICIPI, such as pancreatic swelling and response to corticosteroid treatment [7] bear resemblance to autoimmune pancreatitis (AIP) [8], the term AIP type 3 has been proposed [7]. However, the biological basis for classifying ICIPI as a form of AIP has not yet been established. Moreover, most published reports have relied primarily on clinical, biochemical, and imaging findings, while histopathological data remain limited. Recent studies suggest that ICIPI may encompass a spectrum of histological injury patterns, some of which overlap with established features of AIP [9], but the extent and diagnostic significance of this overlap remain incompletely defined.

It was the aim of our study to describe the clinicopathological and histological features of rare ICIPI cases with available tissue and to critically assess overlap with established histological patterns of AIP in the context of published evidence.

## 2 Patients, Material, and Methods

From the electronic patient record system at Karolinska University Hospital (TakeCare) patients receiving ICI therapy were retrieved. This list was cross‐referenced with the records from the Department of Pathology on pancreatic specimens. Furthermore, a survey was initiated in the pancreatic pathology community via our networks, including the European Pancreatic Club (EPC). Histological specimens were originally diagnosed by expert pancreatic pathologists at participating institutions (CFM, AK, GK, AH, and SR) and subsequently underwent central review by an expert pancreatic pathologist (CSV). The central review included assessment of the extent and distribution of key histopathological findings, including inflammatory cell infiltrates, granulocytic epithelial lesions (GELs) [10], fibrosis (including storiform fibrosis), obliterative phlebitis, and increased numbers of IgG4‐positive plasma cells. Clinical data were collected from the records.

A comprehensive literature search was conducted in PubMed, Google Scholar, and J‐STAGE (Japan Science and Technology Information Aggregator, Electronic) to identify published case reports and patient series describing histopathologically confirmed ICI‐associated pancreatitis. Search queries combined ICI drug names (class‐level and individual agents) with pancreatitis terminology and histopathological descriptors. Pre‐specified inclusion and exclusion criteria were applied systematically to all retrieved records. Eligibility required: (1) original case report or patient series; (2) pancreatitis attributed to ICI therapy; (3) explicit histopathological description from pancreatic tissue obtained by biopsy, surgical resection, or autopsy; and (4) human subjects. Studies reporting only biochemical enzyme elevation without tissue confirmation, and those addressing ICI‐associated new‐onset diabetes without pancreatic inflammatory pathology, were excluded. The search and triage process was performed by JML, CSV, and CFM, with borderline cases resolved by consensus. Results are reported descriptively; formal PRISMA‐compliant systematic review methodology was not applied (Figure S1). The histopathological findings were collated and discussed by a panel of pathologists. To further explore these patterns and formally test their relationship to AIP type 1 and type 2, the pooled histopathological data were also examined using multivariate statistical methods.

For this analysis, the graded features from the own series and literature reports (Tables 1, 2) were pooled at the level of the individual tissue probe (*n* = 25: 19 literature, 6 own; 23 patients, two of whom—Chen et al.'s patient 1 [9] and our Patient 1 (Table 3)—were each sampled twice since they represent two different samples). Acinar‐ductal metaplasia, recorded only in the own series, was excluded from the multivariate analysis and reported descriptively only. Specimen type and data source were retained as reference labels rather than active variables, so that any effect of how tissue was sampled could be distinguished from the underlying disease pattern.

**TABLE 1:** Histological features of the patients with ICIPI.

Specimen type	Patient 1		Patient 2	Patient 3	Patient 4	Patient 5
	EUS‐FNB	Surgical (PDE)	EUS‐FNB	EUS‐FNB	EUS‐FNB	Surgical (PDE)
Inflammatory cells:						
Lymphocytes	++	+++	**+**	**+**	**+**	++
Plasma cells	+	+++	+	**−**	+	+++
Neutrophils	**−**	+	+	**−**	**−**	++
Eosinophils	**−**	**−**	**−**	+	+	−/+
Duct‐centric distribution	−*	+++	−*	−*	−*	+++
GELs	**−**	++	**−**	**−**	**−**	++
Fibrosis	+ (non‐storiform)	+ (non‐storiform)	+ (non‐storiform)	+ (non‐storiform)	+ (non‐storiform)	+ (non‐storiform)
Atrophy	+	+/++	+/++	+	+	+/++
Acinar‐ductal metaplasia	+	+/++	+	**−**	**−**	+/++
Obliterative phlebitis	**−**	**−**	**−**	**−**	**−**	**−**
Increased IgG4+ plasma cells	**−**	**−**	**−**	**−**	**−**	**−**
ICDC:						
AIP1—Level 1						
AIP1—Level 2						
AIP2—Level 1		+				+
AIP2—Level 2						
None	+		+	+	+	

*Note:* −*: medium‐sized ducts not represented. −: absent; +: weakly or focally present; ++: moderately present; +++: strongly present.

Abbreviations: ADM, acinar‐ductal metaplasia; FNB, fine needle biopsy; GELs, granulocytic epithelial lesions; ICDC, international consensus diagnostic criteria for AIP [11]; PDE, pancreatoduodenectomy.

**TABLE 2:** Summary of histological characteristics of published case reports on ICIPI where pathology could be reviewed.

Reference	Specimen type	Inflammation					GELs	Fibrosis		Atrophy	Oblit. Phlebitis	IgG4+ cells
		Lympho‐cytes	Plasma cells	Neutrophils	Eosinophils	Duct‐centric		Present	Storiform			
Chen (*n* = 4) [9]	FNB, autopsy	+/++	+	+/++	−/+	—	−/+	+ (in 3 cases)	+/++ (in 1 case)	+ (in 3 cases)	**−**	**−**
Janssens [12]	FNB	+	**−**	+	**−**	**−**	**−**	+	**−**	+	**−**	**−**
Tanabe [13]	FNB	+	+	**−**	**−**	**−**	**−**	++	**−**	++	**−**	**−**
French [14]	FNB	+	+	**−**	**−**	**−**	**−**	+	**−**	+	**−**	**−**
Hirota [15]	FNB	+	+	++	**+**	**−**	**−**	+	**−**	+	**−**	**−**
Shi [16]	FNB	+	**−**	+	**−**	**−**	**−**	**−**	**−**	+	**−**	**−**
Suda [17]	FNB	+	+	**−**	**−**	**−**	**−**	**−**	**−**	ND	**−**	**−**
El Tawil [18]	FNB	++	++	**−**	**−**	**−**	**−**	++	**−**	—	**−**	ND
Song [19]	FNB	+	**−**	**−**	**−**	ND	**−**	+	**−**	+	**−**	**−**
Ikemoto [20]	FNB	+	**−**	**−**	**−**	**−**	**−**	+	**−**	+	**−**	**−**
Ley [21]	FNB	++	+	+	+	++	**−**	++	**−**	+	**−**	**−**
Rawson [22]	Sx	+	+	+	**−**	**−**	+	++	**−**	+	**−**	**−**
Champion [23]	Sx	+	++	**−**	**−**	++	**−**	**−**	+	+	+	+
Bastens [24]	Sx	++	++	++	**−**	++	**−**	++	**−**	++	**−**	**−**
Inoue [25]	Sx	++	++	**−**	**−**	ND	**−**	++	**−**	+	+	+

*Note:* −: absent; +: weakly or focally present; ++: moderately present; +++: strongly present.

Abbreviations: FNB, fine needle biopsy; GEL, granulocytic epithelial lesion; ICIPI, immune check point inhibitor associated pancreatic injury; ND, not determined; Sx, surgical specimen.

**TABLE 3:** Clinical features of the patients with ICIPI and histology.

Characteristics	Patient 1	Patient 2	Patient 3	Patient 4	Patient 5
Age at diagnosis	56	43	23	45	56
Gender	Female	Female	Female	Female	Male
Underlying disease	Malignant melanoma	Breast cancer	LCH	Malignant melanoma	Malignant melanoma
ICI therapy	Combined therapy	PD‐1/PD‐L1 therapy	Combined therapy	Ipilimumab + Nivolmab	Nivolumab
Estimated time to onset (days)	580	198	182	540	356
Peak lipase (µkat/L)	3.94	12.06	39	11.67	nl
Peak amylase (µkat/L)	0.35	5.35	4.72	nd	nd
IgG‐levels (g/L)	8.23	9.07	4.76	nd	nd
Ref. intervall: 9–15 g/L				nd	0.270
IgG4 (g/L)	0.1	0.28	0.1	nd	
Ref. intervall: 0.05–1.25					
Fecal Elastase‐1 levels (microg/g)	14	346	5	nd	nd
Abdominal pain	No	No	Yes	Yes	No
CT‐abnormalities	Yes swollen, with lesion	Yes swollen	Yes swollen	Yes swollen	Yes lesion (metastasis?)
Type of treatment	Surgical resection	Corticosteroids	Corticosteroids	Corticosteroids	Surgical resection
Outcome	PEI	Pancreatic atrophy	PEI		

Abbreviation: PEI, pancreatic exocrine insufficiency.

To position each probe along a type 1‐versus‐type 2 axis, composite scores were calculated by summing the ordinal grades of the features characteristic of each subtype: a “type 1 score” (storiform fibrosis + obliterative phlebitis + IgG4‐positive plasma cells) and a “type 2 score” (granulocytic epithelial lesions + duct‐centric inflammation + neutrophils). Probes were also grouped by unsupervised hierarchical clustering using Gower's distance (a measure of dissimilarity suited to graded, non‐numeric data) and Ward's method; three clusters were specified in advance, corresponding to the nonspecific, type 1‐leaning, and type 2‐leaning patterns, and their reproducibility was assessed by bootstrap resampling. Two further ordination techniques (principal component and multiple correspondence analysis) served as cross‐checks that the groups were not an artifact of the scoring method. The association between cluster membership and specimen type, and separately with data source, was tested with Fisher's exact test (Monte Carlo simulation given the small cell counts). Given the small sample size, these analyses are exploratory and hypothesis‐generating rather than confirmatory.

The study was approved by the National IRB (EPN Dnr. 2024‐00203‐01).

## 3 Results

### 3.1 Clinical Characteristics and Outcome

We identified five patients with ICIPI and pancreatic histopathology (4 female, one male; age range 23–56 years): endoscopic ultrasound‐guided fine‐needle biopsy (EUS‐FNB) in three cases, surgical specimen in one case, and both biopsy and resection specimen in a further case (Table 3). All patients were diagnosed between 2020 and 2025. Three patients suffered from malignant melanoma requiring ICI therapy, one patient had breast cancer and another patient had Langerhans cell histiocytosis.

Pancreatitis became clinically manifest between 182 and 580 days following the start of ICI. All patients received a combination of different ICI (e.g., ipilimumab plus nivolumab). Peak serum lipase levels ranged between 4 and 41 (µkat/L). Only one patient was symptomatic at presentation; this patient was later found to carry a CFTR mutation. This mutation (c.3454G>C—p.(Asp1152His)) carries only a minor risk of CFTR‐related pancreatic disease [26]. All patients were kept on their ICI treatment due to the necessity for the underlying malignant condition.

**FIGURE 1:**
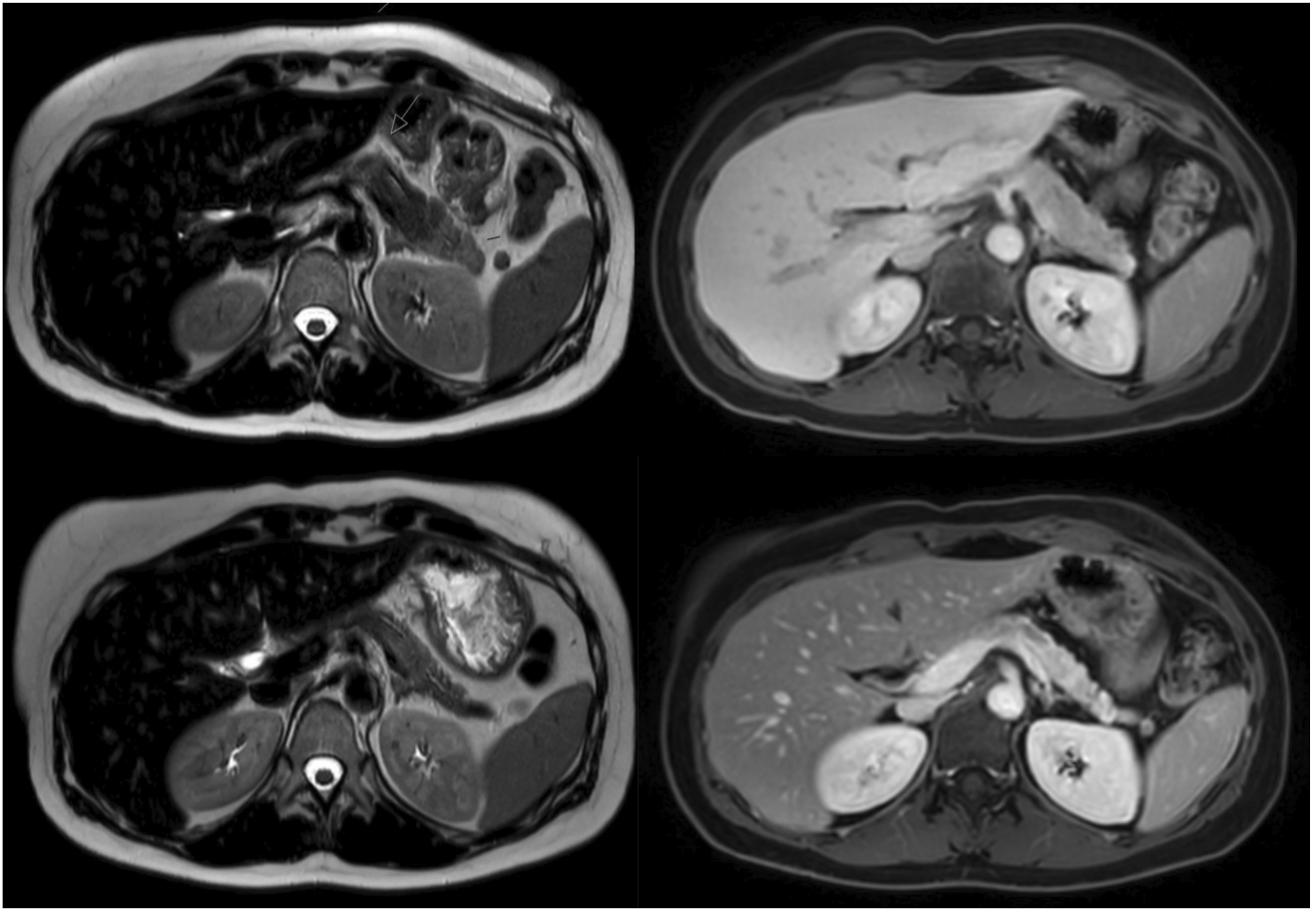
Radiological imaging of immune checkpoint inhibitor‐induced pancreatitis (patient 2). Magnetic resonance images (T2‐weight—left; diffusion‐weight—right) at the time of ICIPI diagnosis (03/2020, upper panel) and 2 months later following steroid therapy (lower panel). The typical image of an autoimmune pancreatitis with swelling of the gland, drawn contures, and vanishing of the main pancreatic duct are obvious as well as improvement after treatment.

On cross‐sectional imaging, the cases demonstrated a swollen pancreas, as described for AIP, and were considered pathognomonic on CT‐scan or MRI investigations (Figure 1). IgG4 levels were measured in three patients and found to be normal. In addition to the ongoing ICI therapy, the three patients who did not undergo surgery received corticosteroid therapy. The dosing and course were in line with the guidelines for AIP [8]. This led to a reduction in pancreatic enlargement (Figure 1) and a decrease in amylase and lipase levels. Fecal elastase‐1, measured in three patients, was very low in two and deteriorated in the third patient (> 800 μg/g, later 346 μg/g), who also demonstrated pancreatic atrophy on imaging and had loose stools. Therefore, pancreatic enzyme replacement therapy was initiated in these three patients. As of spring 2026, none of the patients experienced a recurrence of the ICIPI.

### 3.2 Histology

**FIGURE 2:**
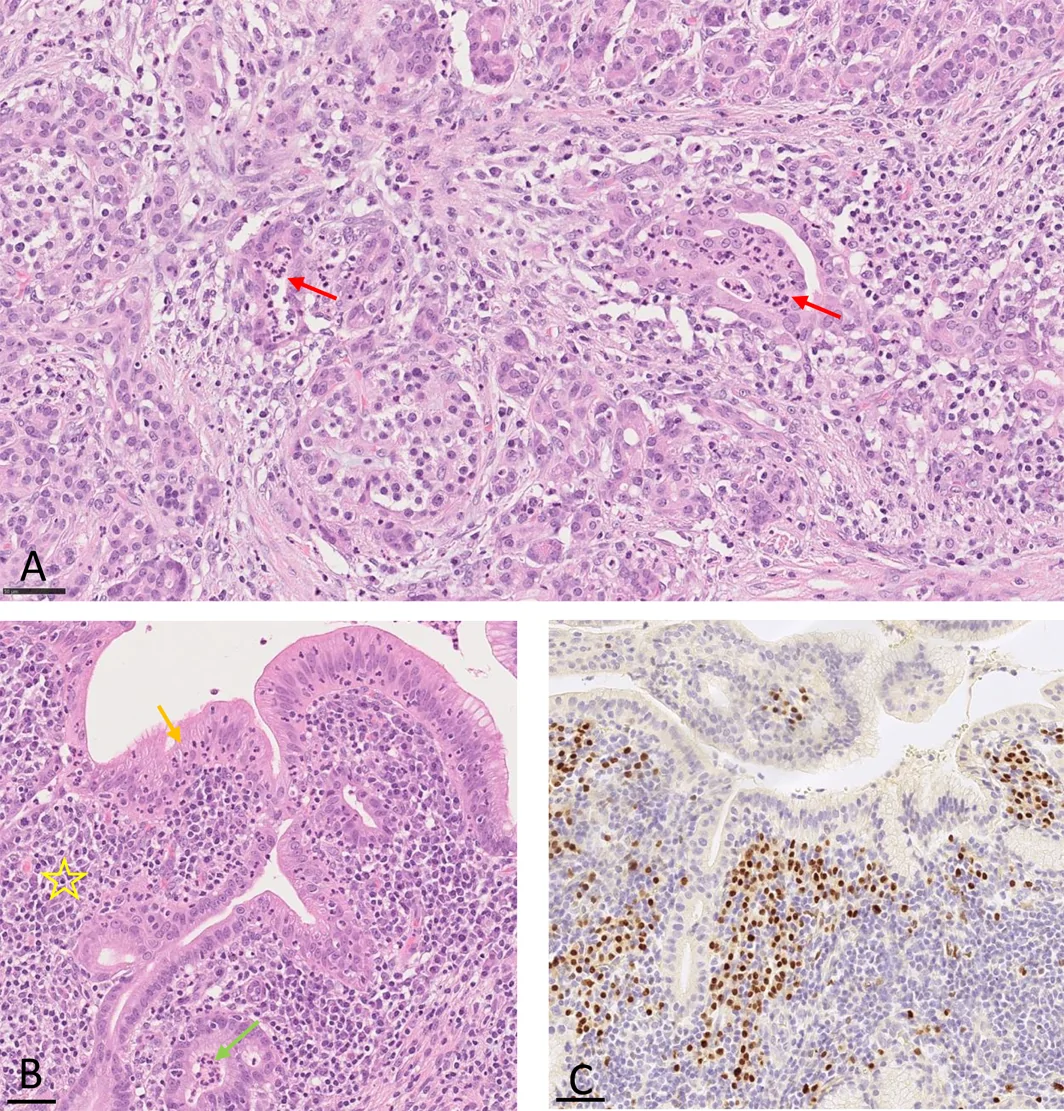
Microscopic images of immune checkpoint inhibitor‐induced pancreatitis (patient 1). Photomicrographs of the patient's surgical resection specimen (pancreatoduodenectomy) showing a mixed inflammatory infiltrate with both lobular (A) and periductal (B, *yellow asterisk*) involvement. The inflammatory process presents numerous granulocytic epithelial lesions (GELs, *red arrows*) with intraepithelial granulocytes (*orange arrow*) and intraluminal microabscesses (green arrow). (C) Immunohistochemical staining with MUM1 (DAB chromogen, brown) and IgG4 (AP chromogen, red) shows no significant increase in IgG4+ plasma cells. Scale bars: (A and B), 100 μm; (C), 250 μm.

Based on the central review and the following panel discussion, consensus was reached between the panel pathologists about the histology of the cases included in the study cohort. Histological findings were similar across all four EUS‐FNBs (22G, Boston Aquire and Covidien SharkCore) and consisted of scant to mild inflammatory cell infiltrates predominantly composed of lymphocytes with only sparse plasma cells (Table 1, Figure 2). The distribution of the infiltration was patchy affecting the acinar parenchyma. Periductal inflammation was not identified; however, only small intra‐ and interlobular ducts were included in the biopsy material. Scattered neutrophils and eosinophils were occasionally observed, but GELs were not observed. The acinar parenchyma showed variable and patchy atrophy associated with non‐storiform fibrosis and occasional foci of acinar‐ductal metaplasia. Overall, findings were nonspecific; none of the biopsies fulfilled the International Consensus Diagnostic Criteria (ICDC) level 1 or 2 for AIP type 1 or 2 [11].

**FIGURE 3:**
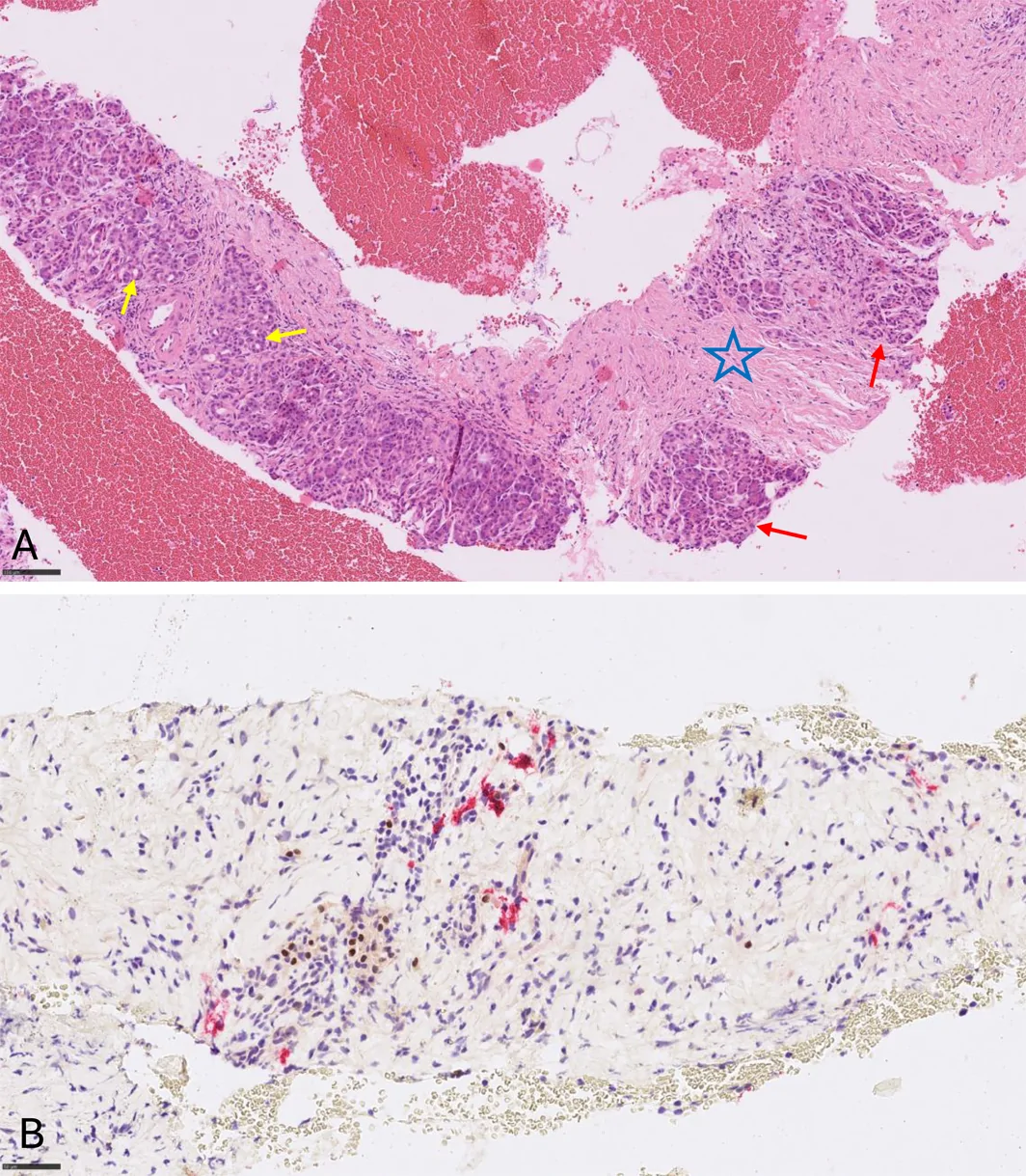
Microscopic images of immune checkpoint inhibitor‐induced pancreatitis (patient 2). Photomicrographs of the patient's fine needle biopsy (FNB) from the tail of the pancreas. (A) Hematoxylin and eosin‐stained section showing a microcylinder of pancreatic tissue with marked fibrosis (*blue asterisk*) and lobular atrophy (*red arrows*), characterized by loss of acinar cells and areas of acinar‐to‐ductal metaplasia (*yellow arrows*). (B) Immunohistochemical staining with MUM1 (DAB chromogen, brown) and IgG4 (AP chromogen, red) shows no significant increase in IgG4+ plasma cells. Scale bars: 250 μm.

In contrast, both surgical specimens showed dense lymphoplasmacytic infiltration with a marked duct‐centric distribution (Figure 3), including the specimen from the patient whose prior EUS‐FNB had yielded only nonspecific changes. In several affected ducts, the lumen was markedly distorted, and the duct‐lining epithelium showed multifocal neutrophilic infiltration consistent with GELs. Despite the abundance of plasma cells, IgG4+ cells were scant. The acinar parenchyma showed varying, overall, relatively mild mixed inflammatory infiltration and patchy atrophy ranging from mild to advanced. Accompanying fibrosis was non‐storiform. Focal non‐obliterative phlebitis was observed in one case. Taken together, these findings formally fulfilled the ICDC level 1 criteria for type 2 AIP [11].

### 3.3 Review of the Literature

In 13 of the 18 patients retrieved from the literature, histological evaluation was based on EUS‐FNB. A common finding across these cases was chronic inflammatory infiltration, predominantly lymphocytic, with only sparse plasma cells (Table 2). Duct‐centric inflammation was reported in only three cases. Atrophy of varying degree was another frequent feature, observed in all but two cases. While fibrosis was reported in the majority of cases, storiform fibrosis was identified in only a single case [9]. Neutrophilic infiltration of the acinar parenchyma was present in half of the cases, whereas GELs, obliterative phlebitis, or increased numbers of IgG4‐positive plasma cells were rarely observed. Overall, none of the cases demonstrated features fulfilling the diagnostic criteria of AIP type 1 or type 2 [11].

In four case reports, histology was based on a surgical specimen [22, 23, 24, 25]. One case lacked histological feature suggestive of AIP type 1 or type 2 and was reported as acute on chronic pancreatitis. Because pancreatitis in this patient was associated with pancreas annulare and histologically described as acute‐on‐chronic pancreatitis [22], a causal relationship with preceding ICI therapy remains uncertain. The inflammatory changes were more likely related to recurrent inflammation in the setting of annular pancreas.

Two cases exhibited a significant increase in IgG4‐positive plasma cells [23, 25], obliterative phlebitis, and—in one case—storiform fibrosis, consistent with AIP type 1. In the fourth case, duct‐centric inflammation and GELs were identified, representing histological features classically associated with AIP type 2 (Table 4). However, in this case, the features were associated with the preceding ICI exposure.

**TABLE 4:** Synopsis of the histological features in AIP type 1 and type 2, and in ICIPI.

Feature	AIP type 1	AIP type 2	ICIPI (*n* = 22)	ICIPI of AIP type (*n* = 8)
Storiform fibrosis	+++	—	2	1
Lymphocytic infiltration	+++	−/(+)	22	8
IgG4+ cells	+++	−/(+)	2 (ND in 2)	2
Obliterative phlebitis	++	**−**	−2	2
Neutrophils	**−**	++	11	7
GEL	**−**	+++	4	3
Pancreatic atrophy	Common	Common	20	8

*Note:* −: absent; +: weakly or focally present; ++: moderately present; +++: strongly present; (+): less commonly weak/focal presence.

Abbreviations: AIP, autoimmune pancreatitis; GEL, granulocytic epithelial lesion; ICIPI, immune check point inhibitor‐associated pancreatic injury; ND, not determined.

### 3.4 Multivariate Analysis of the Histopathological Spectrum

To formally test the impressions gathered from the own series and the literature review, the graded features of both cohorts were pooled and analyzed jointly as described above (25 tissue probes from 23 patients: 19 literature, 6 own). This combined analysis reproduced and sharpened the specimen‐related pattern already apparent on qualitative review. Type 1 markers (obliterative phlebitis, IgG4‐positive plasma cells) were absent in every FNB specimen and present in only 8% of probes overall; the type 2 markers (GELs, duct‐centric inflammation) were similarly rare in FNB specimens (6%) but far more common where more tissue was available, reaching 60%–100% in surgical and autopsy specimens.

**FIGURE 4:**
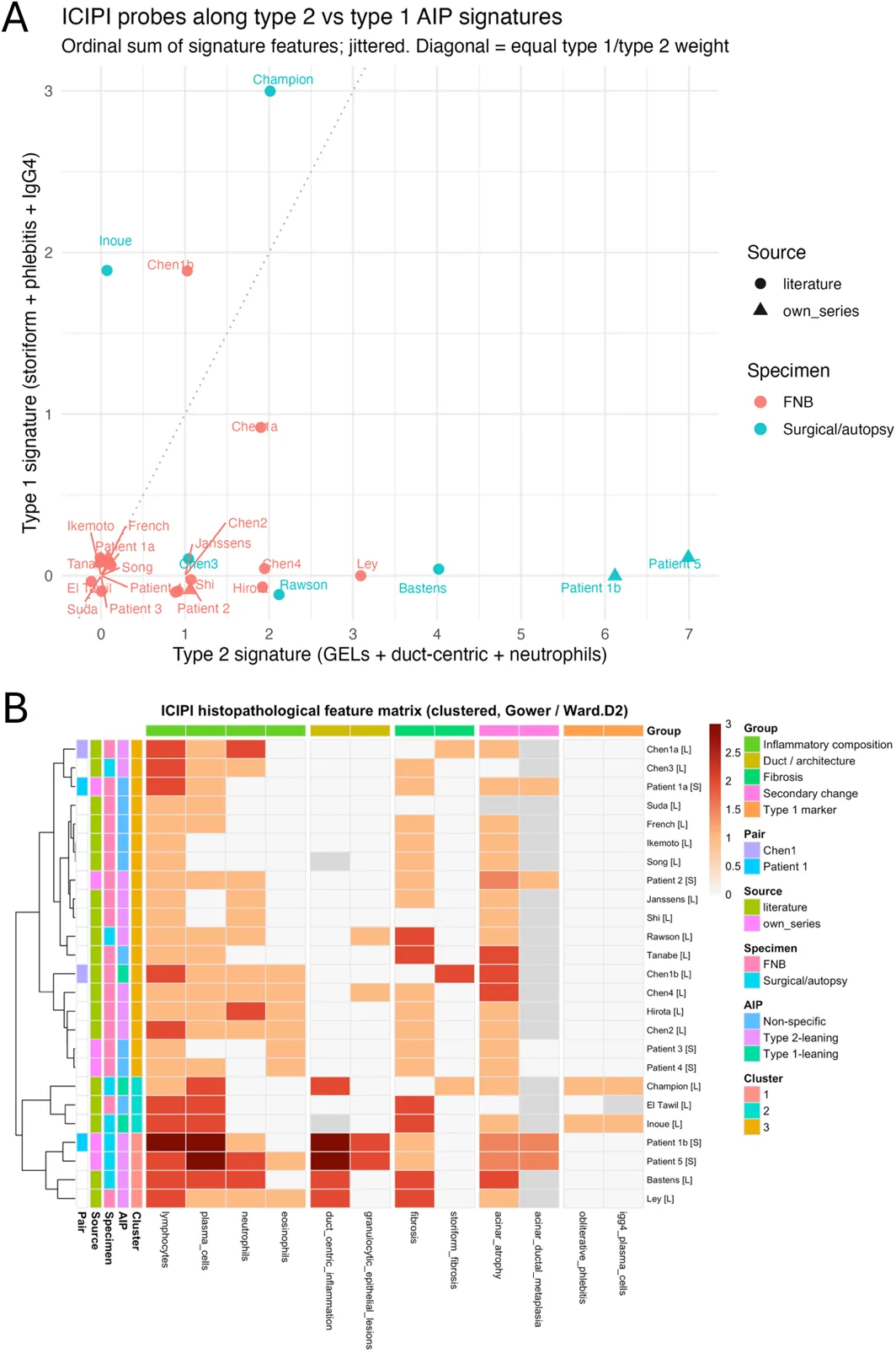
Multivariate analysis of the pooled histopathological feature matrix (25 tissue probes from 23 patients: 19 from the literature, 6 from the own series). (A) Each probe is positioned by its type 2 score (granulocytic epithelial lesion (GELs) + duct‐centric inflammation + neutrophils) and type 1 score (storiform fibrosis + obliterative phlebitis + IgG4‐positive plasma cells)—the sum of the ordinal grades for the features characteristic of each autoimmune pancreatitis (AIP) subtype (see Methods); color denotes specimen type, shape denotes data source. (B) Heatmap of the twelve graded features (color intensity = grade, white = absent to dark red = marked; gray = not recorded), with probes (rows) ordered by unsupervised clustering, which grouped probes by their overall similarity across all features rather than by score alone. Three clusters emerged: nonspecific (cluster 3: 18 probes), type 2‐leaning (cluster 1: 4 probes), and type 1‐leaning (cluster 2: 3 probes). Row annotations indicate assigned cluster, specimen type, data source, and the two probe pairs obtained from the same patient (our Patient 1 and Chen et al.'s patient 1). Acinar‐ductal metaplasia (own series only) is shown for reference but was not used for clustering. Column labels (left to right): lymphocytes, plasma cells, neutrophils, eosinophils, duct‐centric inflammation, GELs, fibrosis, storiform fibrosis, acinar atrophy, acinar‐ductal metaplasia, obliterative phlebitis, and IgG4+ plasma cells. AIP, autoimmune pancreatitis; FNB, fine needle biopsy; GEL, granulocytic epithelial lesion ICIPI, immune check point inhibitor associated pancreatic injury.

Plotting each probe by its type 1 and type 2 composite scores visualized this as a continuum rather than discrete categories: most probes fell near the origin (nonspecific), a subset extended along the type 2 axis and was dominated by resection and autopsy specimens, and a small group extended along the type 1 axis, anchored by the Champion [23] and Inoue [25] cases (Figure 4A). Unsupervised clustering, which groups probes by their overall similarity across all recorded features rather than by a single score, reproduced the same three‐way division and showed good reproducibility on bootstrap resampling: a large nonspecific group (18 probes), a type 2‐leaning group (4 probes, including both of our own resection specimens), and a distinct type 1‐leaning group (3 probes: Champion [23], Inoue [25], and El Tawil [18]) (Figure 4B). Cluster membership was significantly associated with specimen type (Fisher's exact *p* = 0.009) but not with data source (*p* = 0.36), indicating that the histological pattern observed depends on how the tissue was obtained rather than on any systematic difference between the published cases and our centrally reviewed series.

This sampling effect could be observed directly within our own cohort: Patient 1's initial EUS‐FNB grouped with the nonspecific probes, while the resection specimen obtained shortly afterward from the same patient grouped with the type 2‐leaning probes. By contrast, the two biopsies from Chen et al.'s patient 1 [9], obtained using the same technique on separate occasions, remained together in the nonspecific group.

Taken together, these findings indicate that ICIPI encompasses a spectrum of histological manifestations with only partial overlap with type 1 and type 2 AIP, rather than a single reproducible entity, and that the extent of this overlap depends strongly on specimen type.

## 4 Discussion

The increasing use of ICIs has brought to light immune‐related adverse events (ir‐AEs), including pancreatic injury. Although these toxicities are generally considered to be consequences of dysregulated immune activation following checkpoint blockade, the precise biological mechanisms underlying organ‐specific injury remain incompletely understood. In the pancreas, clinical and radiological overlap with AIP has led to the proposal of the term “type 3 AIP” [7, 27]. However, as the exact incidence of ICIPI is unknown but deemed to be very low, histopathological evidence remains limited, and the extent to which ICIPI overlaps with established AIP patterns has not been systematically assessed [2, 28].

Interestingly, in all six surgical specimens, except one reported in the literature, a histological diagnosis compatible with AIP—either type 1 or type 2—could be reached, while this was not the case for any of the biopsy cases, including all EUS‐FNB specimens in our own series. This difference is perhaps not unexpected, given the known limitations of pancreatic biopsy in the histological diagnosis of AIP, where diagnostic accuracy depends on tissue amount, sampling quality, and representation of the relevant inflammatory and fibrosing patterns [29]. In the present series, the limitation is illustrated clearly by Case 1. This is an example of “initially nonspecific” EUS‐FNB, which was shortly thereafter followed by surgery. The specimen obtained from this procedure exhibited pathological features deemed characteristic of AIP type 2. Patients with ICIPI undergoing surgical resection clearly represent a highly selected subgroup, which may have an impact on the histological picture observed in these specimens. First, as outlined above, it is likely to result in enrichment of features—such as GELs—that typically show a patchy distribution. Second, it could be argued that patients who underwent surgery presented with a more advanced stage of ICIPI compared with those who underwent EUS‐FNB, potentially accounting for the nonspecific findings on EUS‐FNB. However, several factors challenge this assumption: first, the considerable variability in the time elapsed from ICI initiation to diagnostic intervention (EUS‐FNB or surgery) and second, the fact that surgical indications were driven by a suspicion of malignancy rather than the clinical severity of the condition.

The report by Chen and colleagues represents, to our knowledge, the first dedicated histology‐based case series of ICIPI and provides an important comparison for our findings [9]. Similar to our cohort, they reported pancreatic enlargement, inflammatory infiltration with neutrophilic components, fibrosis, scant IgG4‐positive plasma cells, and absence of obliterative phlebitis, arguing against a close relationship to type 1 AIP. However, notable differences emerged: compared with the older patients in the Chen series (71–83 years) [9], our cohort was substantially younger (23–56 years). While patient age is determined by the malignant disease that was treated with ICI, it remains unknown whether patient age affects any aspects of ICIPI, including its histological manifestations. Second, the interval from ICI initiation to pancreatic injury tended to be longer. Histologically, their cases were predominantly characterized by acinar‐centric mixed inflammation with only focal GEL‐like duct injury insufficient for a diagnosis of type 2 AIP, whereas our surgical cases showed marked duct‐centric inflammation with definite GELs and stronger overlap with type 2 AIP, including two cases fulfilling ICDC level 1 criteria. This discrepancy may partly reflect sampling differences, as our EUS‐FNB specimens likewise showed only limited, nonspecific changes. Overall, these findings support ICIPI as a heterogeneous histopathological spectrum rather than a single reproducible entity.

The histological changes described in the published case reports vary considerably in terms of inflammatory composition and extent, fibrosis, and duct involvement. While such heterogeneity does not exclude a shared underlying injury mechanism, it makes it difficult to define a single histopathological pattern specific to ICIPI. To date, the most consistent findings are inflammatory infiltrates of varying composition and acinar injury, often accompanied by secondary changes such as acinar‐to‐ductal metaplasia, atrophy, and fibrosis (Table 4). Overall, these features are nonspecific. Unlike type 1 AIP, where a significant proportion of cases may be diagnosed based on histology, ICIPI generally remains dependent on clinical correlation.

What are the factors leading to ICIPI? Apparently, combination of ICI increases the risk of developing irAE in general but a pancreatic affection in particular [6]. Since in one of our patients, the youngest, we later discovered a CFTR mutation, genetic factors prone for the development of a pancreatitis may enhance this risk.

What are the similarities between ICIPI and AIP? First, both conditions may present with pancreatic enlargement on radiological imaging that can be difficult to distinguish (Figure 1 and [30]). Second, ICIPI and AIP may respond to corticosteroid therapy [3, 8]. Third, a subset of cases, particularly those evaluated in surgical specimens, may demonstrate substantial histological overlap with established AIP patterns, particularly type 2 AIP. However, such morphological similarities should not necessarily be interpreted as evidence of a shared pathogenesis or indeed as an indication that ICIPI represents a bona fide subtype of AIP.

The diagnosis of ICIPI is clinically important not only because pancreatitis and abdominal pain may warrant treatment, but also because of the risk of long‐term pancreatic dysfunction and the potential to overlook a primary pancreatic malignancy or metastatic lesion. As illustrated in one of the cases with profound pancreatic atrophy [12], there is a risk of both exocrine and endocrine pancreatic insufficiency. Indeed, all three of our cases with available follow‐up developed pancreatic exocrine insufficiency requiring pancreatic enzyme replacement therapy according to guidelines [31]. Of note, the largest study cohort to date—comprising 248 patients with lipase elevation and 6367 with normal lipase levels—demonstrated a significant reduction in pancreatic volume associated with lipase elevation during the course of ICIPI [30], although pancreatic function was not systematically assessed in that study. To learn about the significance and frequency of pancreatic exocrine insufficiency in ICIPI, follow‐up of patients and assessment of pancreatic exocrine function when clinically relevant is suggested.

In conclusion, ICIPI, an established clinical diagnosis, appears to be associated with a spectrum of histological manifestations rather than a distinct pathognomonic pattern. Based on our own cases and review of the published literature, some cases exhibit substantial overlap with established histological patterns of type 1 or type 2 AIP, whereas others remain nonspecific. Until larger study cohorts will provide better insight into the histological changes associated with ICIPI, and its potential biological relationship with AIP, the descriptive term “ICI‐associated/induced pancreatitis” (ICIP) may be more appropriate than a classification as “type 3 AIP” [32]. It is important to point out that in the vast majority of patients, ICIP is mild and can be treated with steroids while the ICI therapy is ongoing [33]. Follow‐up, as recommended for any kind of newly diagnosed pancreatitis [34, 35], is highly recommended.

## Conflicts of Interest

The authors declare no conflicts of interest.

## Supporting information


**Figure S1:** Flow diagram of the study selection and eligibility assessment process.The chart illustrates the multi‐stage screening pipeline used to identify the currently available literature evaluating pancreatic histopathology in patients with immune check point inhibitor associated pancreatic injury (ICIPI). ICI drug names were entered both on the individual agent level and the class of the ICI drug. Formal PRISMA‐compliant systematic review methodology was not applied.

## Data Availability

Data sharing is not applicable to this article as no datasets were generated or analyzed during the current study.
