# Supplementary material for: Immune Checkpoint Inhibitor‐Induced Pancreatitis
Source: United European Gastroenterol J. 2026 Sep 7;14(7):e70285. doi: 10.1002/ueg2.70285 (PMC13548779; doi:10.1002/ueg2.70285)
Supplement: Supplementary file 1 [file UEG2-14-e70285-s001.docx]

# Supplementary Files

**Supplementary Figure 1:** Flow diagram of the study selection and eligibility assessment process.
The chart illustrates the multi-stage screening pipeline used to identify the currently available literature evaluating pancreatic histopathology in patients with immune check point inhibitor associated pancreatic injury (ICIPI). ICI drug names were entered both on the individual agent level and the class of the ICI drug.

Formal PRISMA-compliant systematic review methodology was not applied.

Abbreviations: ICI = immune check point inhibitor; irAE = immune-related adverse events. T1DM = Type 1 Diabetes mellitus.
